# Cancers of Unknown Primary Origin: Real-World Clinical Outcomes and Genomic Analysis at the European Institute of Oncology

**DOI:** 10.1093/oncolo/oyae038

**Published:** 2024-03-23

**Authors:** Luca Boscolo Bielo, Carmen Belli, Edoardo Crimini, Matteo Repetto, Liliana Ascione, Gloria Pellizzari, Celeste Santoro, Valeria Fuorivia, Massimo Barberis, Nicola Fusco, Elena Guerini Rocco, Giuseppe Curigliano

**Affiliations:** Division of New Drugs and Early Drug Development for Innovative Therapies, European Institute of Oncology, IRCCS, Milan, Italy; Department of Oncology and Hemato-Oncology, University of Milan, Milan, Italy; Division of New Drugs and Early Drug Development for Innovative Therapies, European Institute of Oncology, IRCCS, Milan, Italy; Division of New Drugs and Early Drug Development for Innovative Therapies, European Institute of Oncology, IRCCS, Milan, Italy; Department of Oncology and Hemato-Oncology, University of Milan, Milan, Italy; Early Drug Development Service, Memorial Sloan Kettering Cancer Center, New York, USA; Division of New Drugs and Early Drug Development for Innovative Therapies, European Institute of Oncology, IRCCS, Milan, Italy; Department of Oncology and Hemato-Oncology, University of Milan, Milan, Italy; Division of New Drugs and Early Drug Development for Innovative Therapies, European Institute of Oncology, IRCCS, Milan, Italy; Department of Oncology and Hemato-Oncology, University of Milan, Milan, Italy; Division of New Drugs and Early Drug Development for Innovative Therapies, European Institute of Oncology, IRCCS, Milan, Italy; Department of Oncology and Hemato-Oncology, University of Milan, Milan, Italy; Division of New Drugs and Early Drug Development for Innovative Therapies, European Institute of Oncology, IRCCS, Milan, Italy; Department of Oncology and Hemato-Oncology, University of Milan, Milan, Italy; Division of Pathology, IEO, European Institute of Oncology IRCCS, Milan, Italy; Department of Oncology and Hemato-Oncology, University of Milan, Milan, Italy; Division of Pathology, IEO, European Institute of Oncology IRCCS, Milan, Italy; Department of Oncology and Hemato-Oncology, University of Milan, Milan, Italy; Division of Pathology, IEO, European Institute of Oncology IRCCS, Milan, Italy; Division of New Drugs and Early Drug Development for Innovative Therapies, European Institute of Oncology, IRCCS, Milan, Italy; Department of Oncology and Hemato-Oncology, University of Milan, Milan, Italy

**Keywords:** cancer of unknown primary, treatment options, genomic profiling, immunotherapy

## Abstract

**Background:**

Cancer of unknown primary origin (CUP) poses a significant challenge due to poor clinical outcomes and limited treatment options. As such, further definition of clinicopathological factors and genomic profile to better adapt treatment strategies is required.

**Methods:**

Medical records were interrogated to retrospectively include CUP with available clinical and genomics data at the European Institute of Oncology. Next-generation sequencing (NGS) included targeted panels. Statistical analyses were conducted with R Software 4.2.2.

**Results:**

A total of 44 patients were included. With a median follow-up of 39.46 months (interquartile range [IQR] 35.98-47.41 months), median PFS (mPFS) to first-line regimen was 3.98 months (95% CI 3.22-5.98), with a clinical benefit rate of 26% (95% CI 14%-49%), and disease control rate (DCR) limited to 48.28%. Most patients (26 of 31, 83.87%) received platinum-doublet chemotherapy, with no statistically significant difference between first-line treatment regimens. Median OS (mOS) was 18.8 months (95% CI 12.3-39.9), with a 12-month OS rate of 66% (95% CI 50%-85%). All patients received comprehensive genomic profiling (CGP). For 11 patients, NGS was unsuccessful due to low sample quantity and/or quality. For the remaining, TP53 (*n* = 16, 48%) and KRAS (*n* = 10, 30%) represented the most altered (alt) genes. No microsatellite instability was observed (0 of 28), while 6 of 28 (21.43%) tumors carried high TMB (≥10 mutation per megabase). Eight of 33 tumors (24.2%) displayed at least one actionable alteration with potential clinical benefit according to ESCAT. Only 2 of them received targeted therapy matched to genomic alterations, with a combined mPFS of 2.63 months (95% CI 1.84-not evaluable) as third-line regimens. Six patients received anti-PD1/PD-L1 therapy, showing a meaningful mPFS of 13 months (95% CI 2.04-not evaluable).

**Conclusion:**

CUP exhibits poor prognosis with limited benefits from standard treatment regimens. A significant proportion of CUPs carry actionable alterations, underscoring the importance of genomic profiling to gather additional treatment opportunities. In addition, immunotherapy might represent a valuable treatment option for a subset of CUP. Finally, accurate definition of sequencing methods and platforms is crucial to overcome NGS failures.

## Teaser

This study investigated the clinical outcomes of patients affected by cancers of unknown primary in a real-world context and examined whether comprehensive genomic profiling might provide clinical utility by addressing biomarker identification to possibly adapt personalized and targeted treatment strategies.

**Implications for Practice**

The results of this study provide evidence concerning the clinical outcomes of cancers of unknown primary origin (CUPs) in a real-world context, underscoring the limited benefits yielded by standard regimens in the treatment of this category of tumors. In addition, an outline of the genomic landscape of CUPs is provided, assessed with comprehensive genomic profiling by next-generation sequencing, to highlight the significant proportion of CUPs harboring potentially actionable genomic alterations. Lastly, evidence is provided regarding the potential clinical utility achieved through the use of targeted therapy matched to molecular alterations and with the use of immunotherapy, which might deliver additional treatment opportunities to ultimately yield improved patient-centric outcomes.

## Introduction

Cancer of unknown primary origin (CUP), encompassing cancers of unidentified primary origin, represents 2% of all malignant solid tumor diagnosis^1^ and establish an unmet clinical need due to poor long term clinical outcomes.^2^

Approximately half of CUP present as adenocarcinomas of unknown primary site (ACUPs), characterized by mucin production, tubule formation, and immunohistochemistry (IHC) findings that define the histologic subtype without pinpointing the exact origin.^2^ The remaining comprises a diverse group of nonadenocarcinomas (non-ACUPs), encompassing squamous cell carcinomas, neuroendocrine carcinomas, and undifferentiated carcinomas.^2^

Despite extensive diagnostic efforts, involving various imaging modalities, invasive procedures and serum biomarker tests, identifying the primary tumor site remains elusive in a significant number of cases. Currently, there is a paucity of drugs and regimens specifically approved for CUP treatment. Existing therapeutic strategies usually involve multiagent cytotoxic chemotherapy, resulting in a generally poor prognosis with a 1-year survival rate of 20%.^2^ As such, an unmet clinical need exists in defining additional clinical and molecular biomarkers to better adapt treatment strategies accordingly.

In this context, the present study aims to investigate the clinical outcomes of patients affected by CUP in a real-world context and to examine whether CGP might provide clinical utility by addressing biomarker identification to possibly adapt personalized and targeted treatment strategies.

## Methods

### Patient Selection

We conducted a retrospective, single-center observational study to include patients affected by CUP at the European Institute of Oncology. Inclusion criteria consisted in the diagnosis of CUP, for which clinical data and comprehensive genomic profile (CGP) were available. CPG included targeted, tumor-only NGS platforms (FoundationOne CDx, Oncomine Comprehensive Assay v3, and Archer FUSIONPlex). Clinical data with follow-up were extracted from electronic medical reports.

Tumor histotypes were allocated in the ACUP group to include CUP exhibiting adenocarcinoma histological features, and in the non-ACUP group to include squamous cell carcinomas, neuroendocrine carcinomas, and undifferentiated carcinomas. Neuroendocrine carcinomas were included in the study, regardless of last ESMO recommendation,^2^ as the timeframe of consecutive patients included the study ranged before this latter publication, and as such were labelled as CUP as per previous ESMO guidelines,^3^ for which NGS was performed according to the European Institute of Oncology’s (IEO) Molecular Tumor Board (MTB) recommendation.^4^

In the genomic analysis, genomic variants were filtered to include only pathogenic and likely pathogenic alterations, and annotated according to the original report classification. Clinical actionability was defined according to the ESMO Scale for Clinical Actionability of molecular Targets (ESCAT),^5^ and potential clinical benefit was considered for genomic alterations included as ESCAT evidence tier I (alteration-drug match associated with improved outcome in clinical trials) to tier III (alteration-drug match suspected to improve outcome based on clinical trial data in other tumor type or with similar molecular alteration). Tumor mutational burden was categorized as high versus low using a cutoff of ≥ 10 mut/Mb, as previously reported.^6^

### Endpoints and Statistical Analysis

The primary aim of the study was to investigate mPFS, defined from the start of the first-line treatment for locally advanced or metastatic disease to progression or death, whichever occurred first; and mOS, calculated as the time from the start of first-line treatment for the locally advanced or metastatic disease to death.

Other clinical endpoints of interest included mPFS in patients receiving immune checkpoint inhibitors (ICIs) and in patients receiving matched molecular therapy (MMT), this latter defined as molecular targeted therapy administered according to an actionable genomic variant detected in CGP.

Continuous variables were reported as median and interquartile range or mean and standard error, as appropriate, and categorical variables as numbers and proportions and compared using the Chi-square test or Fisher’s exact test, as appropriate.

Median follow-up time was calculated using the reverse Kaplan-Meier method.

The Kaplan-Meier method was used to estimate survival curves, and time-to-event endpoints calculated using log-rank statistics to account for censored data.

Non-adjusted hazard ratios for single variables were calculated using the Cox regression model. Adjusted hazard ratios in the multivariable model were calculated if at least 2 covariates were found to be statistically significant in the univariable model. Variables were included as covariate in the Cox regression model if demonstrated to satisfy the proportional hazards assumption. Variables included in the univariable and multivariable Cox regression model for PFS and OS included age as a continuous variable, sex, number of metastatic sites (<2; ≥2), presence of liver metastasis, presence of brain metastasis, histotype category (ACUP vs. non-ACUP), and type of first-line regimen (platinum-based doublet chemotherapy vs. non-platinum-based doublet chemotherapy).

Hypothesis tests and survival analysis were conducted assuming a statistical significance with an alpha level of < 0.05.

Statistical analyses were performed using R software version 4.2.2.^7^

The research was approved by the IEO internal review board (IRB) and was conducted in accordance with the principles stated in the Declaration of Helsinki and with the principles of good clinical practice.

## Results

### Patients Characteristics

From February 2017 to May 2023, a total of 44 consecutive patients affected by CUP and treated at the European Institute of Oncology were included in the study. List of baseline clinicopathological characteristics is reported in Table 1. The median number of metastatic sites was 2 (range 1-6), with the most common involved site represented by lymph nodes (*n* = 31, 70.5%), followed by liver (*n* = 16, 36.4%) and lung (*n* = 10, 22.7%). Six patients (13.6%) presented with disease limited to lymph nodes. Seventeen of 44 patients (38.6%) were classified as favorable risk CUP according to the last ESMO guidelines.^2^ Of those, 10 belonged to the single metastatic deposit or oligometastatic disease category, 3 to the renal-like CUP, 1 to the ovary-like CUP, 2 to the head and neck-like CUP, and 1 to the colon-like CUP.^2^

**Table 1.** Patients baseline characteristics.

Characteristic	*N* = 44^1^
Age	59 (21-80)
*Sex*	
Female	23/44 (52%)
Male	21/44 (48%)
*Histotype*	
Adenocarcinoma	22/44 (50%)
Undifferentiated carcinoma	13/44 (30%)
Neuroendocrine carcinoma	5/44 (11%)
Squamous carcinoma	4/44 (9%)
*Category*	
ACUP	22/44 (50%)
Non-ACUP	22/44 (50%)
*Risk category*	
Unfavourable risk	33/44 (61%)
Favourable risk	17/44 (39%)
*Metastatic sites no. (%)*	
<2	11/44 (25%)
≥2	33/44 (75%)
Liver metastasis (%)	16/44 (36%)
Brain metastasis (%)	3/44 (7%)
Previous surgery	10/44 (23%)

^1^Median (minimum-maximum); *n*/*N* (%).

Abbreviations: *N*, number; ACUP, adenocarcinomas of unknown primary site; no., number.

### Genomics

All 44 patients received CGP with a tissue based, tumor-only, targeted NGS. For 11 of 44 (25%) patients the analysis was unsuccessful due to low-quality and quantity sample material. For those, sampled site consisted in soft tissue (*n* = 4), lymph-nodes (*n* = 2), liver (*n* = 2), bone (*n* = 1), peritoneal (*n* = 1), while for one patient sample site was unknown. For the remaining with successful sequencing, lymph node and liver represented the 2 most common sampled sites used for genomic analysis (*n* = 13, 39.39%; and *n* = 8, 24.24%, respectively).

Tumor mutational burden (TMB) and microsatellite status was available for 28 patients. Median TMB was 4 (IQR 0.75-8), with 6 of 28 (21.43%) tumors categorized as TMB-high. No microsatellite instability was observed (0 of 28).

**Figure 1.**
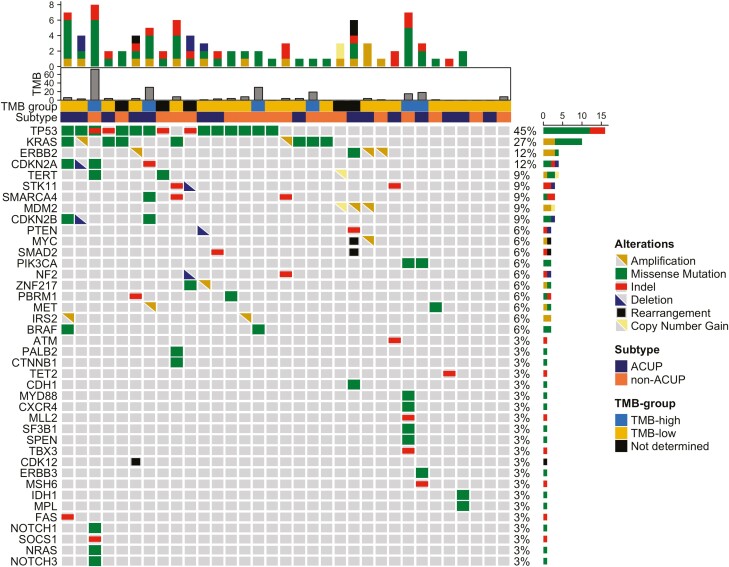
Oncoprint of oncogenic and likely oncogenic alterations. Abbreviations: Indel, insertion-deletion; ACUP, adenocarcinomas of unknown primary site; Non-ACUP, nonadenocarcinomas of unknown primary site. Oncoprint reports the most common oncogenic and likely oncogenic alterations found in the whole cohort of tumors, further labelled by CUP histological subtype, Tumor Mutational Burden group, and absolute Tumor Mutational Burden value. The top 40 most common altered genes were selected arbitrarily.

The most commonly altered gene was TP53 (15 of 33, 45%), followed by KRAS (9 of 33, 27%). List of genomic variants along with alterations class is reported in Fig. 1.

Concerning alterations (alt) in the TP53 pathway (*CDKN2A*, *MDM2*, *MDM4*, *TP53*), RTK/Ras/PI3K/AKT signaling (*EGFR*, *ERBB2*, *PDGFRA*, *MET*, *KRAS*, *NRAS*, *HRAS*, *BRAF*, *NF1*, *SPRY2*, *FOXO1*, *FOXO3*, *AKT1*, *AKT2*, *AKT3*, *PIK3R1*, *PIK3CA*, *PTEN*), and RB pathway (*CDKN2A*, *CDKN2B*, *CDKN2C*, *CDK4*, *CDK6*, *CCND2*, *RB1*), we observed a similar frequency in ACUP compared to non-ACUP for all signaling pathways (TP53 pathway^alt^ 8 of 22, vs. 10 of 22, respectively, *P* = .76; RTK pathway^alt^ 10 of 22 vs. 13 of 22, *P* = .547; RB pathway^alt^ 2 of 22 vs. 7 of 22, *P* = .132).

Actionable genomic alterations with potential clinical benefits according to ESCAT were observed in 8 of 33 (24.2%) patients. These included ERBB2 amplification (*n* = 3), ERBB2 D769 (*n* = 1), KRAS G12C (*n* = 1), BRAF V600E (*n* = 1), MET amplification (*n* = 1), IDH1 R132C (*n* = 1). Actionable alterations were observed in tumors showing histotype features of adenocarcinoma (*n* = 3), undifferentiated carcinoma (*n* = 3), and neuroendocrine carcinoma (*n* = 1).

### Clinical Outcomes

Of 44 patients, 33 received systemic treatment for locally advanced and metastatic disease. For those, first-line regimens included platinum-based doublet (26 of 31, 83.87%), non-platinum doublet (2 of 31, 6.45%), platinum-based triplet (1 of 31, 3.23%), avelumab (1 of 31, 3.23%), and sunitinib (1 of 31, 3.23%). Considering platinum-based doublets, most patients received taxanes with platinum-salts (12 of 26, 46.15%), followed by gemcitabine (8 of 26, 30.77%), etoposide (5 of 26, 19.23%), and anthracycline (1 of 26, 3.85%).

**Figure 2.**
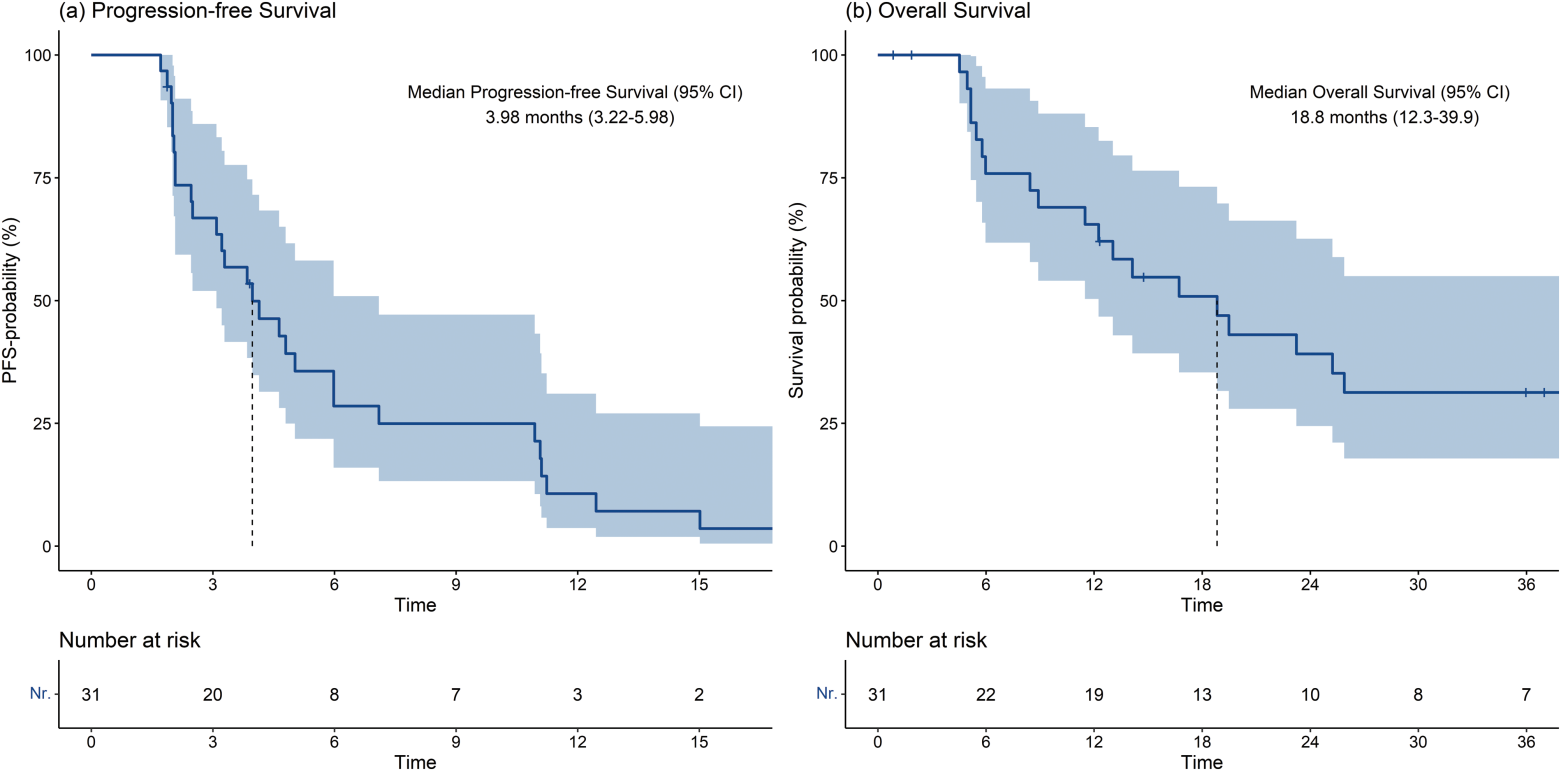
Kaplan-Meier plots of progression-free survival (**a**) and overall survival (**b**). Abbreviations: PFS-probability, progression-free survival probability.

With a median follow-up of 39.46 months (interquartile range [IQR] 35.98-47.41 months), median PFS (mPFS) on the first-line treatment was 3.98 months (95% CI 3.22-5.98), with a clinical benefit rate of 26% (95% CI 14%-49%; Fig. 2a). An overall response rate (ORR) of 27.59% emerged, with a disease control rate limited to 48.28%. In the univariable analysis both the type of regimen (*P* = .901) and the type of platinum doublet (*P* = .8) did not significantly associate with PFS. In the univariable analysis, no other individual covariate associated with PFS (Supplementary Fig. S1).

In the overall population, median OS (mOS) was 18.8 months (95% CI 12.3-39.9), with a 12-month OS rate of 66% (50-85%; Fig. 2b). In the univariable model, only male sex significantly associated with OS (HR 3.52, 95% CI 1.35-9.20, *P* = .01; Supplementary Fig. S2).

Ten patients (10 of 44, 22.7%) underwent curative-intent surgery. Of those, one patient received adjuvant chemotherapy and 2 adjuvant radiation therapy. Six patients (6 of 10, 60%) demonstrated disease recurrence, with a median disease-free survival of 3.47 months (0.75-not evaluable). Patients undergoing surgery demonstrated higher mOS compared to patients who did not (HR 0.22, [0.06-0.77], *P* = .01).

Patients showing alterations in TP53 (11 of 31, 35.48%) and KRAS (5 of 31, 16.13%) did not demonstrate different mOS compared to TP53 wild type (wt) and KRAS wt tumors (HR 1.50 [0.61-3.66], *P* = .37 TP53; HR 2.18 [0.71-6.66], *P* = .17 KRAS] (Supplementary Fig. S3). Similarly, no difference was observed in TMB-high (5 of 28, 17.8%) compared to TMB-low tumors (HR 0.91 [0.30-2.71], *P* = .9).

Six patients received ICI in the metastatic setting, distributed across various treatment lines: 1 as a first-line regimen, 4 as a second-line, and 1 as a fifth-line regimen. Of them, 3 were affected by adenocarcinoma, 2 by undifferentiated carcinoma, and 1 by neuroendocrine carcinoma. Tumors exposed to ICI demonstrated a high median TMB of 24.5 mut/Mb (IQR 12.4-36.6), and selection for ICI therapy was based for 5 of such cases upon IEO’s MTB recommendation according to such biomarker as an off-label treatment. For one of the 6 patients, affected by pelvic and lymph nodes metastatic involvement, avelumab as first-line regimen was granted upon the expression by the tumor of histotypes and biomarkers features suggestive yet not conclusive of Merkel cell carcinoma. Overall, a meaningful mPFS of 13 months (95%CI 2.04-not evaluable) was observed in patients subjected to ICI treatment. For the patient receiving avelumab as a first-line regimen, a PFS of 2.0 months was observed. Of note, this latter exhibited a low-TMB of 8 Mut/Mb.

Two of 8 (25%) patients whose tumors showed actionable genomic variants received MMT. In detail, one patient with adenocarcinoma harboring a missense point mutation in IDH1 (R132C) received ivosidenib as a third-line treatment, resulting in a progression-free survival (PFS) of 3.4 months. Another patient, diagnosed with undifferentiated carcinoma with a BRAF V600E alteration, underwent trametinib treatment as a third-line regimen, showing a PFS of 1.8 months.

## Discussion

Cancers of unknown primary remain a major clinical challenge, exhibiting poor prognosis with limited treatment opportunities. In the present study, we reported the challenging outcomes in treating those tumors in a real-world context, exhibiting an mOS of 18.8 months with a limited 12-month OS rate of 66%.

For CUP, treatment strategies are usually adapted according to baseline clinicopathological factors. CUP presenting with single-site or oligometastatic disease are generally managed with local therapy when amenable, irrespective of histology and anatomical site.^2,8^ In our study, 22.7% of patients underwent curative intent surgery, with those showing a longer mOS compared to tumors who did not (mOS 40.9 months vs. 13.6 months), thus confirming the superior prognosis of oligometastatic CUP amenable to local ablative treatments.

Conversely, for patients presenting with advanced disease, platinum-based chemotherapy is generally recommended.^2^ Yet, limited benefits are observed from first-line platinum-based regimens, with no additional benefit resulting from triplet-chemotherapy regimens compared to doublet combinations.^2,9^ Similarly, in the present study, we observed an mPFS limited to 3.98 months to first-line regimens, with 51.72% of patients showing progressive-disease as best response. Moreover, as previously reported,^2^ we observed no difference in efficacy between different platinum-based regimens, thus emphasizing the limited efficacy of conventional chemotherapy regimens in the management of CUP.

To overcome a limited one-size fits-all approach, studies using a site-specific definition to direct treatments personalization have been conducted. A study using microarray-based analysis to employ a site-specific therapy did not result in significant improvements compared to an empirical paclitaxel plus carboplatin regimen.^10^ In another single-arm phase II study, an NGS-based algorithm was used to define the alleged site of origin to adapt chemotherapy regimens, which resulted in an encouraging mPFS of 5.2 months with an mOS of 13.7 months, thus showing encouraging outcomes for such an approach.^11^

Besides its potential use to define the putative-site of origin by defining site-specific genomic variants, NGS represents an appealing opportunity to gather additional treatment options by detecting actionable genomic variants. In this context, previous reports underscored the significant frequency of actionable alterations in CUP, reported to range from 30% to 72% according to the level of actionability considered.^11-15^ In our study, we observed 24.2% of CUP to harbor at least one actionable alteration. Of note, we regarded those as alterations with potential clinical benefit according to the ESCAT scale, thereby reducing the number of patients whose tumors carried hypothetically actionable alterations.

Furthermore, in order to adapt molecular screening diagnostics, previous reports showed a distinctive distribution of signaling pathways alterations across different CUP histotypes. In particular, a higher frequency of RTK alterations in ACUP compared to non-ACUP was reported,^15,16^ with these latter instead associated with aberrations in cell-cycle control and DNA-damage response genes.^16^ Conversely, in our study, we observed non-ACUP to harbor a numerically higher frequency of alterations in TP53 (45.4% vs 36.3%), RTK (59.0% vs. 45.5%) and RB (31.8% vs. 9.0%) pathways. However, we observed actionable alterations to be balanced between ACUP (*n* = 3) and non-ACUP (*n* = 4), thereby not suggesting an histotype-based approach to guide the use of CGP might deliver superior benefits and cost-effective strategies.

Despite no data currently supports the use of MMT as a standard treatment option in CUP, different studies have been conducted to assess its clinical utility. In a single-center study, 15 of 54 CUP harboring at least one actionable alteration received MMT, showcasing variable responses ranging from 1 month to 14 months.^12^ In another study, among 111 CUP, 5 patients harbored EGFR pathogenic variants for which afatinib was administered, with 2 of them showing durable responses for longer than 6 months.^11^ In our study, only 2 of 8 patients harboring actionable alterations received MMT. The limited mPFS of 2.63 months observed in our study could be in part explained by its use as a later line option, with both patients receiving MMT in the third-line setting. Moreover, diverse molecular alterations might yield different benefits for MMT when used in a histo-agnostic manner, as previously reported.^12^ Accordingly, given the high frequency of actionable genomic variants in CUP along with limited efficacy of current standard treatment regimens, further investigation to test the use of MMT to treat CUP is warranted. Nevertheless, drawbacks exist in the applicability of MMT in CUP. Their heterogeneous landscape, arising from diverse tissues and sites, poses a challenge for MMT due to differences in tumor biology, with potential same targets yielding diverse actionability and thus efficacy.^17,18^ Additionally, limitations regarding the approval of drugs specifically for CUP raises concerns about the access to MMT, emphasizing the necessity for inclusion in basket trials exploring MMT irrespective of histology.

In addition to MMT, immunotherapy might represent a valuable treatment option. Previous studies reported a meaningful efficacy of ICI in 56 CUP.^19^ In our study, 6 patients received ICI, for whom in 5 cases the treatment indication was provided upon the detection of a high TMB by NGS assessment. Of note, a durable mPFS of 13 months was observed, thus highlighting the potential efficacy of CUP for a subgroup of patients. In addition, the efficacy of ICI has been described to be higher in CUP expressing PD-L1,^19^ as occurs in approximately 30% of the cases,^20^ as well in tumors showcasing a TMB above 7.75 mut/Mb.^19^ In our study, no PD-L1 testing was conducted for patients who received ICI. Instead, tumors exposed to ICI treatment displayed a significant median TMB of 24.5 mut/Mb. Noteworthy, in our study, one patient affected by a squamous CUP which carried a TMB of 72 mut/Mb, with microstallite stability, received carboplatin plus paclitaxel in the first-line setting, with a limited mPFS of 1.9 months, before showing a long-lasting response to pembrolizumab as a second-line regimen for 31.8 months. As such, considering ICI efficacy in CUP was observed regardless of the putative site of origin,^19^ immunotherapy should be regarded as a valuable treatment option, and particularly in patients whose tumors demonstrate known biomarker of ICI fficacy, such as PD-L1 and for CUP showing high TMB, as observed in our study and as previously reported.^21,22^

Importantly, in our study, we observed a significant number of unsuccessful NGS, with failed reports which accounted for 25% of the cases. Failure in sequencing reports could arise from a variety of pre-analytical, analytical and post-analytical factors, with most arising from sampling procedure yielding low quantity and quality material,^23^ as occurred in our study. Thereby, addressing accurate tissue collection represent a key aspect to optimize the use of CGP. To tackle tissue samples limitations, liquid biopsies may offer a valuable alternative, whose clinical utility to improve patients outcomes for those showing actionable alterations through cfDNA assessment has been previously reported.^24^ Yet, issues related to pre-analytical factors specific for liquid biopsies, including tumor burden and circulating tumor DNA (ctDNA) shedding, may also arise,^25^ demanding a collaboration with molecular tumor boards for the selection of proper sequencing methods and platforms in a case specific manner.^4^

Finally, it must be acknowledged our study presents some limitations: the retrospective nature of the study, which could have led to selection bias; the limited sample size portending limited statistical power; and the lack of complementary pathological biomarkers, such as immunohistochemistry, to integrate CGP with NGS.

## Conclusion

Cancers of unknown primary continue to pose a significant clinical challenge, displaying poor prognosis with limited efficacy from existing standard regimens. This study underscores the prevalent incidence of actionable genomic alterations in CUP, emphasizing the pivotal role of NGS in serving as a crucial tool for revealing biomarkers with potential clinical actionability. Additionally, alternative biomarkers may offer insights into the efficacy of different treatment modalities, including immunotherapy, as occurred in our study with long lasting responses in tumors showcasing biomarkers of ICI efficacy. Lastly, the poor outcomes of CUP further underscore the need to better adapt treatments based on clinical, pathological and molecular biomarkers, extending beyond a one size fits all strategy to achieve better clinical outcomes for this challenging subgroup of tumors.

## Supplementary Material

oyae038_suppl_Supplementary_Figures_S1-S3

## Data Availability

The data underlying this article will be shared on reasonable request to the corresponding author.
