# Supplementary material for: Cancers of Unknown Primary Origin: Real-World Clinical Outcomes and Genomic Analysis at the European Institute of Oncology
Source: Oncologist. 2024 Mar 23;29(6):504–10. doi: 10.1093/oncolo/oyae038 (PMC11145013; doi:10.1093/oncolo/oyae038)
Supplement: oyae038_suppl_Supplementary_Figures_S1-S3 [file oyae038_suppl_supplementary_figures_s1-s3.zip › oyae038_suppl_Supplementary_Figures_S1-S3/Supplementary Figure Captions.docx]

**Supplementary Figure Captions**

Supplementary Figure S1. Univariable Cox-regression model of progression-free survival for baseline characteristics. Abbreviations: HR, hazard ratio; 95%CI, 95% confidence interval; ACUP, adenocarcinomas of unknown primary site.

Supplementary Figure S2. Univariable Cox-regression model of overall survival for baseline characteristics. Abbreviations: HR, hazard ratio; 95%CI, 95% confidence interval; ACUP, adenocarcinomas of unknown primary site.

Supplementary Figure S3. Kaplan-Meier plots of Overall survival according to TP53 and KRAS alterations. Abbreviations: Alt, altered; CI, confidence interval.
