# Supplementary figures and images for: Cancers of Unknown Primary Origin: Real-World Clinical Outcomes and Genomic Analysis at the European Institute of Oncology
Source: Oncologist. 2024 Mar 23;29(6):504–10. doi: 10.1093/oncolo/oyae038 (PMC11145013; doi:10.1093/oncolo/oyae038)

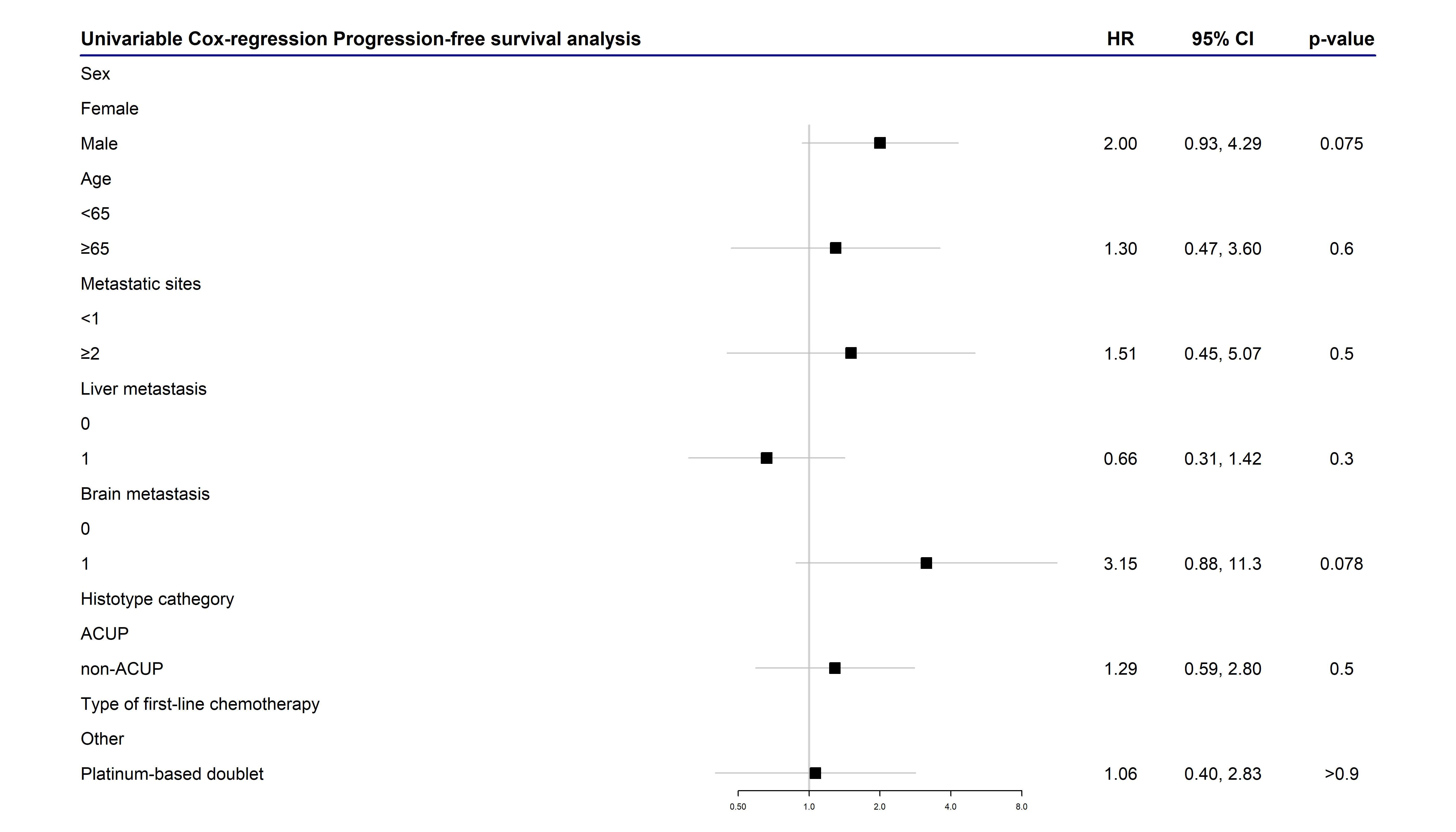

Supplement: oyae038_suppl_Supplementary_Figures_S1-S3 [file oyae038_suppl_supplementary_figures_s1-s3.zip › oyae038_suppl_Supplementary_Figures_S1-S3/Supplementary S1.png]

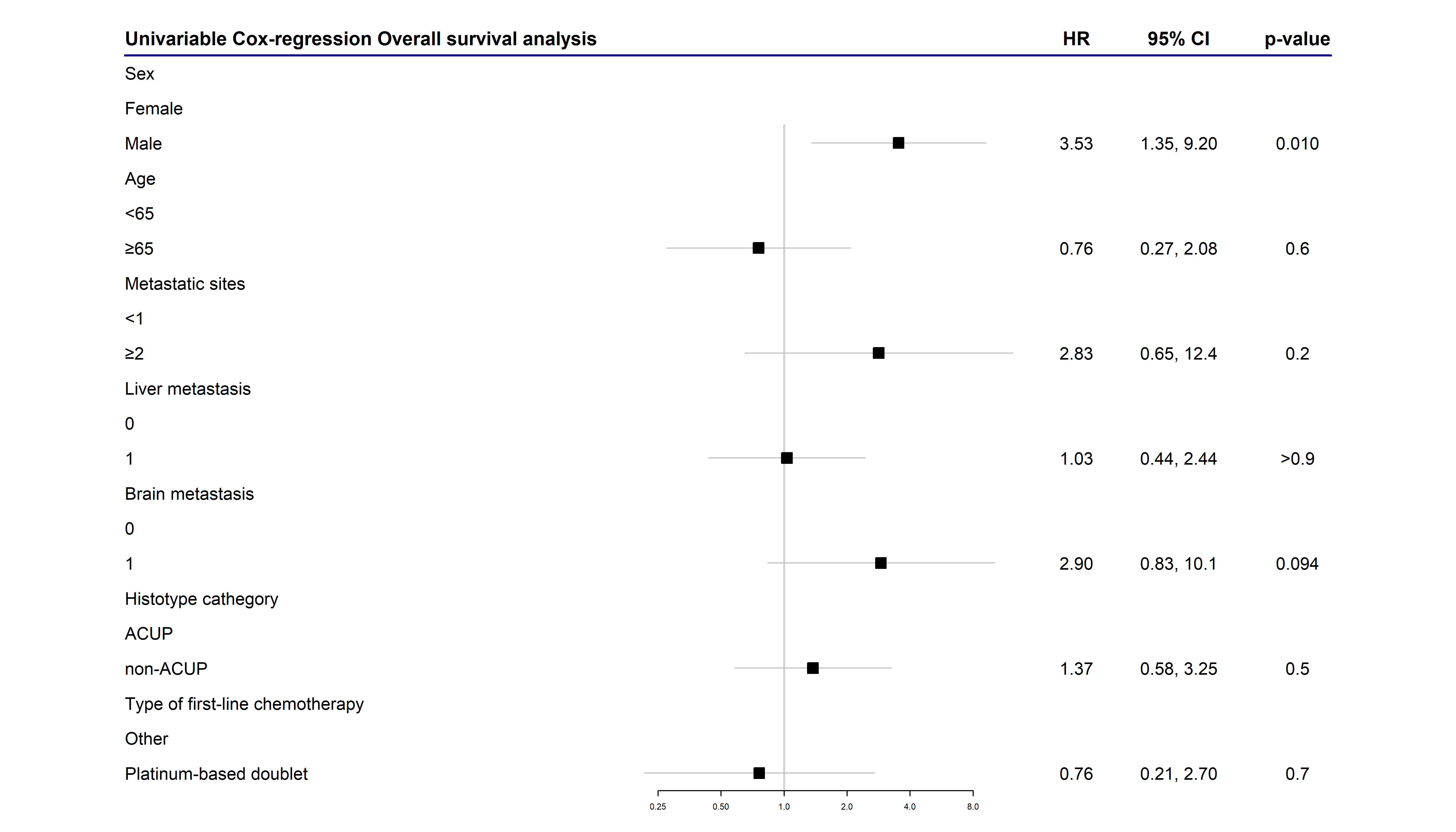

Supplement: oyae038_suppl_Supplementary_Figures_S1-S3 [file oyae038_suppl_supplementary_figures_s1-s3.zip › oyae038_suppl_Supplementary_Figures_S1-S3/Supplementary S2 (2).png]

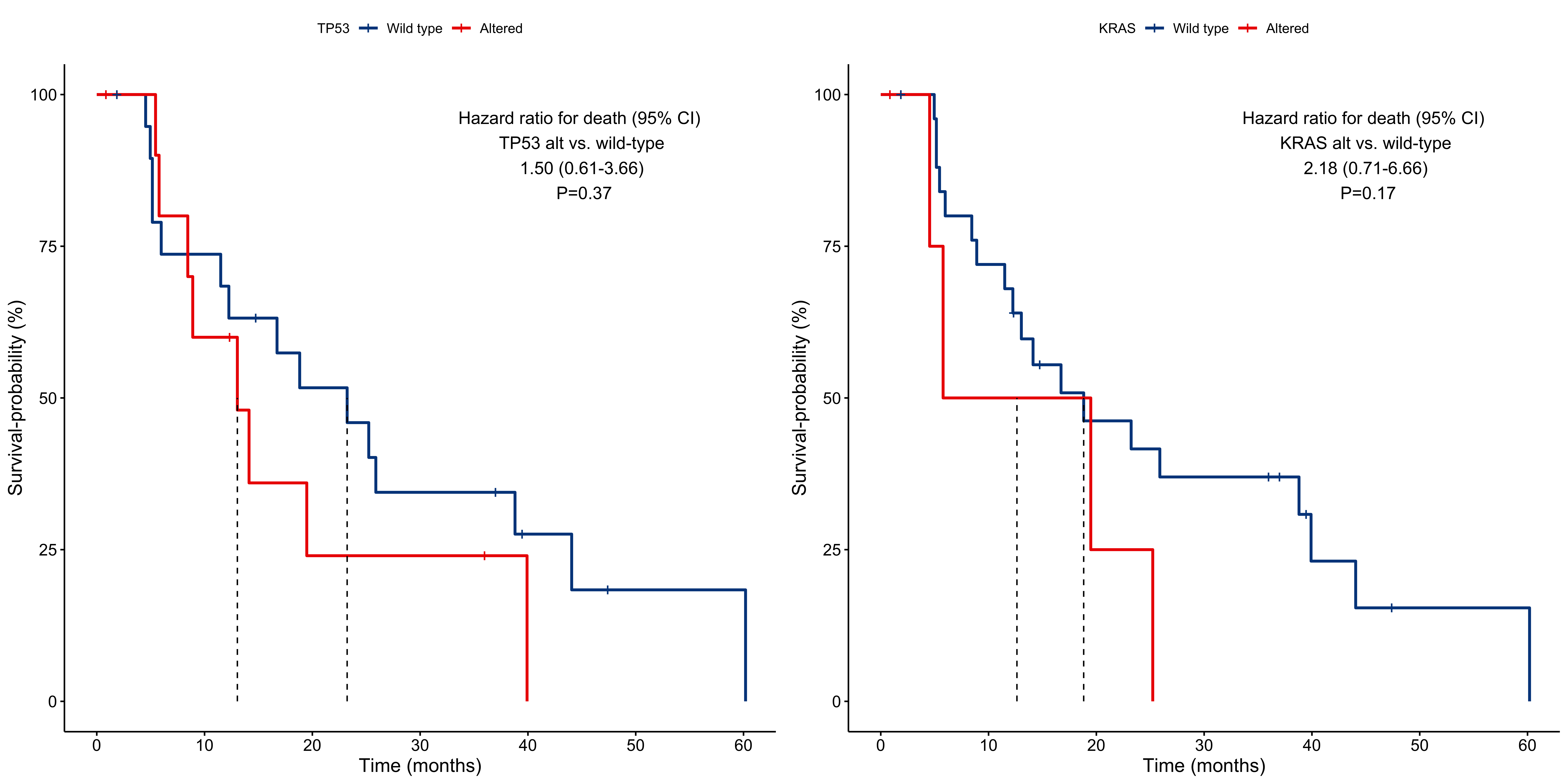

Supplement: oyae038_suppl_Supplementary_Figures_S1-S3 [file oyae038_suppl_supplementary_figures_s1-s3.zip › oyae038_suppl_Supplementary_Figures_S1-S3/Supplementary S3.png]
